# Analysis of the Current Risk of *Leishmania infantum* Transmission in Greece and its Projection

**DOI:** 10.1155/tbed/1087533

**Published:** 2025-07-13

**Authors:** Iván Rodríguez-Escolar, Rodrigo Morchón, Elias Papadopoulos, Georgios Sioutas, Manuel Collado-Cuadrado, Elena Infante González-Mohino, Alfonso Balmori-de La Puente

**Affiliations:** ^1^Zoonotic Diseases and One Health Group, Biomedical Research Institute of Salamanca (IBSAL), Centre for Environmental Studies and Rural Dynamization (CEADIR), University of Salamanca, Salamanca, Spain; ^2^Laboratory of Parasitology and Parasitic Diseases, School of Veterinary Medicine, Faculty of Health Sciences, Aristotle University of Thessaloniki, Thessaloniki, Greece

**Keywords:** canine leishmaniasis, dogs, ecological niche models, Greece, infection risk map, *Leishmania infantum*, *Phlebotomus perfiliewi*, *Phlebotomus tobbi*

## Abstract

Canine leishmaniasis is a vector-borne disease caused by the protozoan parasite *Leishmania infantum*. It is transmitted by different species of the genus *Phlebotomus*, with *Phlebotomus perfiliewi* and *Phlebotomus tobbi* being the most widely distributed species in Greece. Ecological niche models (ENMs) are ecoinformatics tools that have already been successfully applied to model risk maps of other parasitosis according to the environmental variables necessary for their survival. The resulting risk map in this study determines the risk of *L. infantum* infection in Greece, considering the habitat suitability of *Ph. pefiliewi* and *Ph. tobbi*, as well as the infection rate of the parasite in the vector. Central Macedonia, Thessaly, Attica, and islands such as the Cyclades, Dodecanese, and Crete, as well as some urban areas of Thrace, present a high risk of infection. These areas are characterised by a suitable habitat for both vectors and a high infection rate. Regarding the projection to 2080 under a climate change scenario, an increase in transmission risk (9.6%) can be observed in the Epirus area, Peloponnese peninsula, and Aegean islands, occurring mainly towards higher altitude areas. The use of ENMs to generate habitat suitability models of *Ph. perfiliewi* and *Ph. tobbi*, as well as their weighting with the infection rate, is a very useful tool to predict the risk of *L. infantum* transmission in the study area. Thanks to this study, a more complete risk map is obtained that facilitates the control and prevention of this disease.

## 1. Introduction

Leishmaniasis caused by the protozoan parasite *Leishmania infantum* is a zoonotic vector-borne disease that affects both animals and humans, with dogs being the primary domestic host [1]. Its vectors belong to different species of the *Phlebotomus* genus, with *Phlebotomus perfiliewi* and *Phlebotomus tobbi*, being the most widely distributed species in Greece [2].

When feeding on the blood of an infected definitive host, sand flies ingest amastigotes (the tissue form), which then transform into promastigotes (the infective form) in the vector's gut and finally migrate to the proboscis, awaiting the next bite to transmit the parasite to a new host [3]. This parasite development process is temperature-dependent, with the percentage of *L. infantum*-infected vectors increasing logarithmically within the sand fly's survival range (between 10 and 30°C) [4].

Regarding its distribution, leishmaniasis is a cosmopolitan and dynamic disease, both spatially and temporally, influenced by multiple environmental and social factors. In Europe, the Mediterranean Basin countries (such as Greece, Italy, Spain, and Portugal) are endemic regions, with *L. infantum*-associated canine leishmaniasis being much more prevalent than human leishmaniasis [5]. In Greece, reported seroprevalence rates in dogs vary depending on the analyzed region, ranging from 0% to 53%. The overall seroprevalence of *L. infantum*-positive dogs was 13.8% in 2020 with 1265 dogs spread across the country, with the highest prevalences found in the geographical regions of Macedonia (62%), Thrace (34%), and Central Greece (23%) [6].

Two of the most promising approaches for the prevention and control of zoonotic parasitic diseases are geographic information systems (GIS) and ecological niche models (ENMs). Both are ecoinformatics tools that enable disease distribution modeling based on bioclimatic and environmental variables essential for parasite survival. ENMs calculate habitat suitability values for species in a given territory by correlating the organism's distribution points with the environmental variables it responds to [7, 8]. Niche modeling has already been applied to estimate the potential infection risk of zoonotic diseases and validated using presence data of infected hosts [9–11] and potential disease vectors [12, 13].

In the case of leishmaniasis, these tools have been used in the Mediterranean Basin and other regions worldwide to develop infection risk models based on environmental and bioclimatic variables such as temperature, precipitation, vegetation, and human footprint [14–23].

Local studies in Greece have assessed *Leishmania spp*. infection risk in different areas using GIS and ENMs. One study used GIS to analyze the disease in hares from Thessaloniki and Chalkidiki (Macedonia), concluding that leishmaniasis involves a complex interaction between the pathogen, sand flies, environmental influences on vector distribution, and susceptible human and animal populations. This highlights the need for simultaneous investigation of these factors to design effective control and prevention strategies [24]. Another study conducted an ENM analysis of *Leishmania spp*. in Thessaly (Central Greece), identifying that the highest percentage of high-risk infection areas were located in low-altitude irrigated agricultural zones. Additionally, 20% of human settlements were found to be in high-risk areas [25]. However, this study did not integrate an ENM of the vectors with the parasite's development within them, limiting the full potential of these tools in approaching real-world conditions.

Modeling parasite development in vectors, including their potential distribution and multivector models, has made infection risk maps a fundamental methodology for complementing vector-borne zoonotic disease control plans [23, 26–30].

This study aimed to develop a potential infection risk map for canine leishmaniasis in Greece by integrating ENMs of two disease vectors (*Ph. tobbi* and *Ph. perfiliewi*) and the *L. infantum* infection rate in the vector.

## 2. Materials and Methods

### 2.1. Study Area

**Figure 1:**
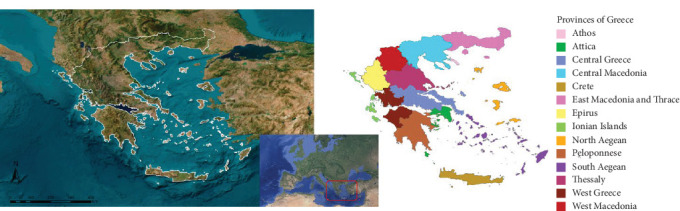
Geographical location of Greece in Europe and the continental, peninsular, and island provinces that comprise it.

Greece (39° 0′ 0” N, 22° 0′ 0” E) (Figure 1), located in southeastern Europe at the crossroads of Europe, Africa, and Asia, was established as the study area. It shares borders to the north with North Macedonia and Bulgaria, the northwest with Albania, and the northeast with Turkey. Greece is part of the Mediterranean basin, with the Aegean Sea bordering its more than 13,676 km of coastline to the east, the Ionian Sea to the west, and the Mediterranean Sea to the south. Greece consists of three types of natural zones: continental, peninsular, and insular. The insular part includes seven archipelagos (the Northern Aegean Islands, Cyclades, Ionian Islands, Dodecanese, Sporades, Saronic Islands, and Crete), with a total of 1400 islands, accounting for 25% of the country's total territory. Crete, located in the Mediterranean Sea, is the largest island, covering 8,331 km^2^. The continental area is characterized by mountain ranges that reach elevations of up to 2917 m at the highest point. The peninsular region is connected to the mainland in the south by the Isthmus of Corinth. In the northeast, the Chalkidiki Peninsula extends southeast into the Aegean Sea. Regarding the country's climate, Greece has a Mediterranean climate with hot, dry summers and mild, wet winters. The mountainous regions experience an alpine climate, mainly in the western part of the country and in the Peloponnese Peninsula, where the average monthly temperature does not exceed 10°C. In the north, a temperate climate prevails, characterized by hot, dry summers and cold, and wet winters [31].

### 2.2. Ocurrence Data

Presence points of the two most widely distributed sand fly vector species of *L. infantum* in Greece, *Phlebotomus perfiliewi* and *Phlebotomus tobbi*, were obtained from different sources, including the widest reported distribution obtained between 1999 and 2004 from the mainland and some of its islands [2], and the European Network for Medical and Veterinary Entomology (VectorNet) of the European Centre for Disease Prevention and Control (ECDC) until present [32]. Once collected, the data were overlaid with a 1 km^2^ grid to account for only one observation per square and reduce spatial autocorrelation biases in the abundance and distribution of observations. At the end of the process, 104 points of *Ph. perfiliewi* and 124 of *Ph. tobbi* were obtained for use in the model.

### 2.3. Bioclimatic and Environmental Data

Fifteen of the 19 bioclimatic variables related to temperature and precipitation were downloaded from the WorldClim database [33] for both current conditions and projections for 2080 [34]. The four excluded variables (BIO_8_, BIO_9_, BIO_18_, and BIO_19_) combined temperature and precipitation data. The next step was to perform a multicollinearity analysis on the bioclimatic variables using the Pearson correlation coefficient in R software [35]. To improve model calibration and avoid cross-correlation between variables, only those with a correlation lower than 0.8 (*r* < 0.8) were included [36]. The selected bioclimatic variables were: BIO_1_ - annual mean temperature, BIO_2_ − mean diurnal range [mean of monthly (max temp − min temp)], BIO_4_ − temperature seasonality (standard deviation × 100), and BIO_12_ − annual precipitation. Regarding environmental variables, vegetation variables (herbaceous and shrub density) were downloaded from EarthEnv [37], and human pressure data (built environment, population density, electric power infrastructure, farmland, grazing land, roads, railways, and waterways) were sourced from the Socioeconomic Data and Applications Center [38]. These variables were selected because of their use in previous work estimating the risk of infection for leishmaniasis. Population density, farmlands, or vegetation are factors that can influence the establishment of vector populations or disease reservoirs [23–25]. All variables were processed in ArcMap 10.8 to ensure a consistent extent (Greece), resolution (1 km^2^ per pixel), and coordinate system (GCS_WGS_1984).

### 2.4. Ecological Niche Model

Habitat suitability models for the two sand fly species (*Ph. perfiliewi* and *Ph. tobbi*) were developed using MaxEnt [39] via the Kuenm package [36] in R software [35]. MaxEnt is an algorithm based on the maximum entropy principle, which assigns habitat suitability values for species in a given territory based on environmental variables [39]. The default MaxEnt settings were used, which include 10000 random background points within the area defined by the layers (Greece) and a logistic output format. Kuenm built 119 models for each species using the following parameter combinations: 17 regularization multiplier values “M” (0.1–1.0 at 0.1 intervals, 2–6 at intervals of 1, 8, and 10), 7 possible combinations of three feature classes “F” (linear, quadratic, and product: l, q, p, lq, lp, qp, lqp), and a single set of variables. The performance of the generated models was assessed considering significance (partial ROC) with 100 iterations and 50% of the data used for bootstrapping, omission rates (OR = 5%), and model complexity (Akaike Information Criterion – AIC). The models were validated with the mean ratio of the area under the curve (AUC), obtained with independent presence points from calibration (20% for testing and 80% for training). The best resulting model (final model) was built using the extrapolation by clamping option, generating 10 replicates based on the same parameter combination selected during calibration. The model was further evaluated using the same criteria described above.

### 2.5. Leishmania infantum Infection Rate in Phlebotomine

The infection rate (% of *Ph. perfiliewi* and *Ph. tobbi* infected by *L. infantum*) was calculated following Rioux et al. [4] and Rodríguez-Escolar et al. [23]. The result was fitted to an ascending logarithmic curve, allowing us to determine the infection rate within the 10–30°C temperature range, which corresponds to the interval where the parasite survives and replicates inside *Ph. ariasi* [4]. This process was conducted in R software [35].

### 2.6. Leishmania infantum Infection Risk Map and its Validation

To generate the infection risk maps, the following steps were necessary: (1) generate the ENMs of *Ph. perfiliewi* and *Ph. tobbi* in raster layers; (2) multiply (weight) the ENMs of both vectors in different proportions to obtain a series of models that simultaneously account for the habitat suitability of both sand fly species; (3) weight the resulting ENMs with the infection rate in Greece to obtain infection risk maps; and (4) validate the resulting maps using georeferenced points of *L. infantum*-infected dogs [6] to select the final risk map that best corresponds to reality. The suitability maps of both vectors (*ENM Ph. perfiliewi⁣*^*∗*^ ENM *Ph. tobbi*) were weighted in different proportions (*Ph. perfiliewi - Ph. tobbi* (PT); 0-100, 25-75, 50-50, 75-25, 100-0). Since each raster pixel contains the probability of sand fly presence, the new combined risk value for each pixel in the resulting models was calculated using the adapted formula [30]:

  $$\begin{array}{c} \text{ENM weighted}=\frac{\text{ENMp}*P+\text{ENMt}*T}{100}, \end{array}$$

where ENMp and ENMt represent the habitat suitability models for *Ph. perniciosus* and *Ph. tobbi*, respectively, and PT corresponds to their percentage contribution to the final model. The resulting models with different proportions were then weighted with the infection rate in Greece, generating five potential risk maps. To validate these risk maps, we used the ArcMap classification method called Natural Jenks (breaks) with five risk classes (“Very High,” “High,” “Medium,” “Low,” and “Very Low”). We then represented the percentage of geolocated infected dogs [6] in histograms for each class to determine which risk map provided the best prediction.

### 2.7. Forward Projection (2080) and Rank Change Analysis

Two ENMs for both sand fly species were generated using MaxEnt [39] with previously selected parameters (multipliers, feature classes, and contribution of each vector to the model), incorporating projections of bioclimatic variables for the period 2061–2080 (2080). The RCP 8.5 scenario, which represents high CO_2_ emissions in Europe [40], was used, with projections from the HadGEM3-GC31-LL model [41] to study the effects of climate change. This climate model is global and is used for future projections. It is one of the most widely used and evaluated general circulation models (GCMs) in ecological niche modeling studies. This model has been shown to adequately capture important climate patterns for Europe. Furthermore, it is among the best-performing models on the continent [42, 43]. Additionally, the infection rate of vectors under the future scenario was calculated using BIO_1_ (annual mean temperature) for 2080. Both present and future maps were converted into binary presence/absence maps using the 10th percentile threshold of the current map to analyze risk change due to *L. infantum* infection under climate change projections. This analysis calculated the percentage of pixels that gained or lost infection risk for the 2080 projection compared to the present map, using the *biomod2* package in R software [44].

## 3. Results

### 3.1. Habitat Suitability Models of *Ph. perfiliewi* and *Ph. tobbi*

**Figure 2:**
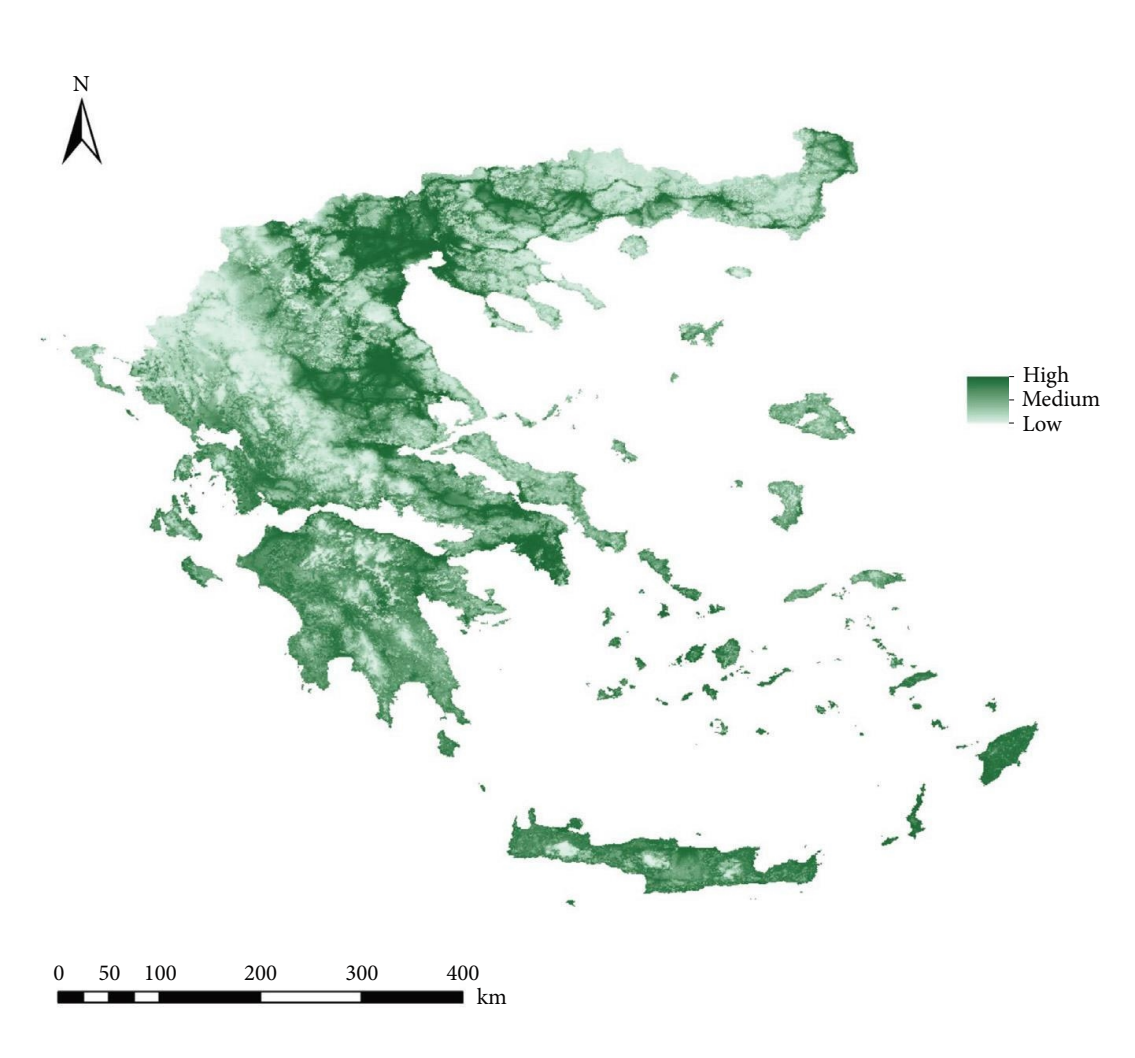
Habitat suitability map (ecological niche model) for *Phlebotomus perfiliewi* in Greece.

**Figure 3:**
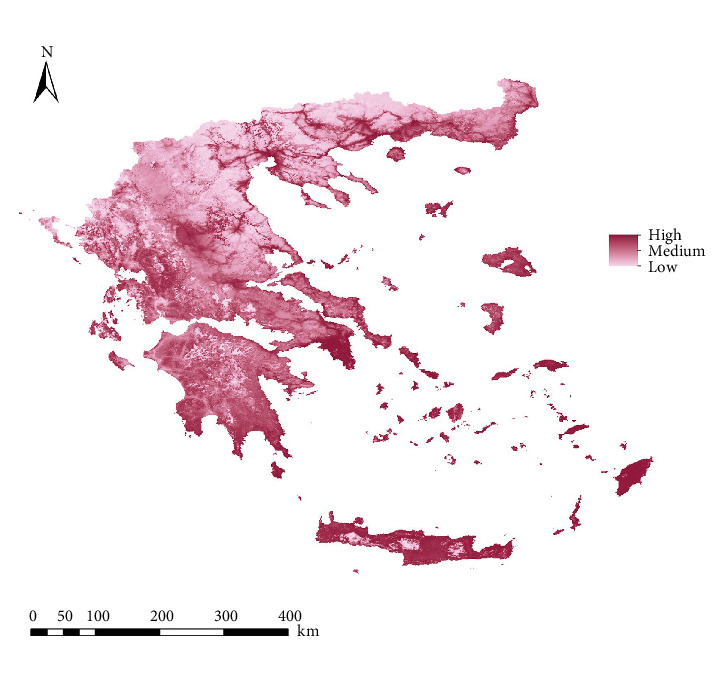
Habitat suitability map (ecological niche model) for *Phlebotomus tobbi* in Greece.

For *Ph. perfiliewi*, out of the 119 models generated, the M_0.7_F_qp model was selected as the best according to Kuenm criteria, with an AUC of 0.839. This model was used to create the final habitat suitability maps (Figure 2). For *Ph. tobbi*, the M_0.2_F_lqp model was selected as the best among the 119 models created, with an AUC of 0.875. Finally, this model was also used to generate the final habitat suitability maps (Figure 3).

The percentage contribution of the selected variables in the suitability models varied between the two species. For *Ph. perfiliewi*, the variables contributed from 3.9% (Herbaceous) to 55.7% (Human footprint), while for *Ph. tobbi*, they ranged from 3.6% (Herbaceous) to 33.2% (BIO_1_) (Table 1).

### 3.2. Map of Leishmania infantum Infection Rate in Ph. perfiliewi and Ph. tobbi

**Figure 4:**
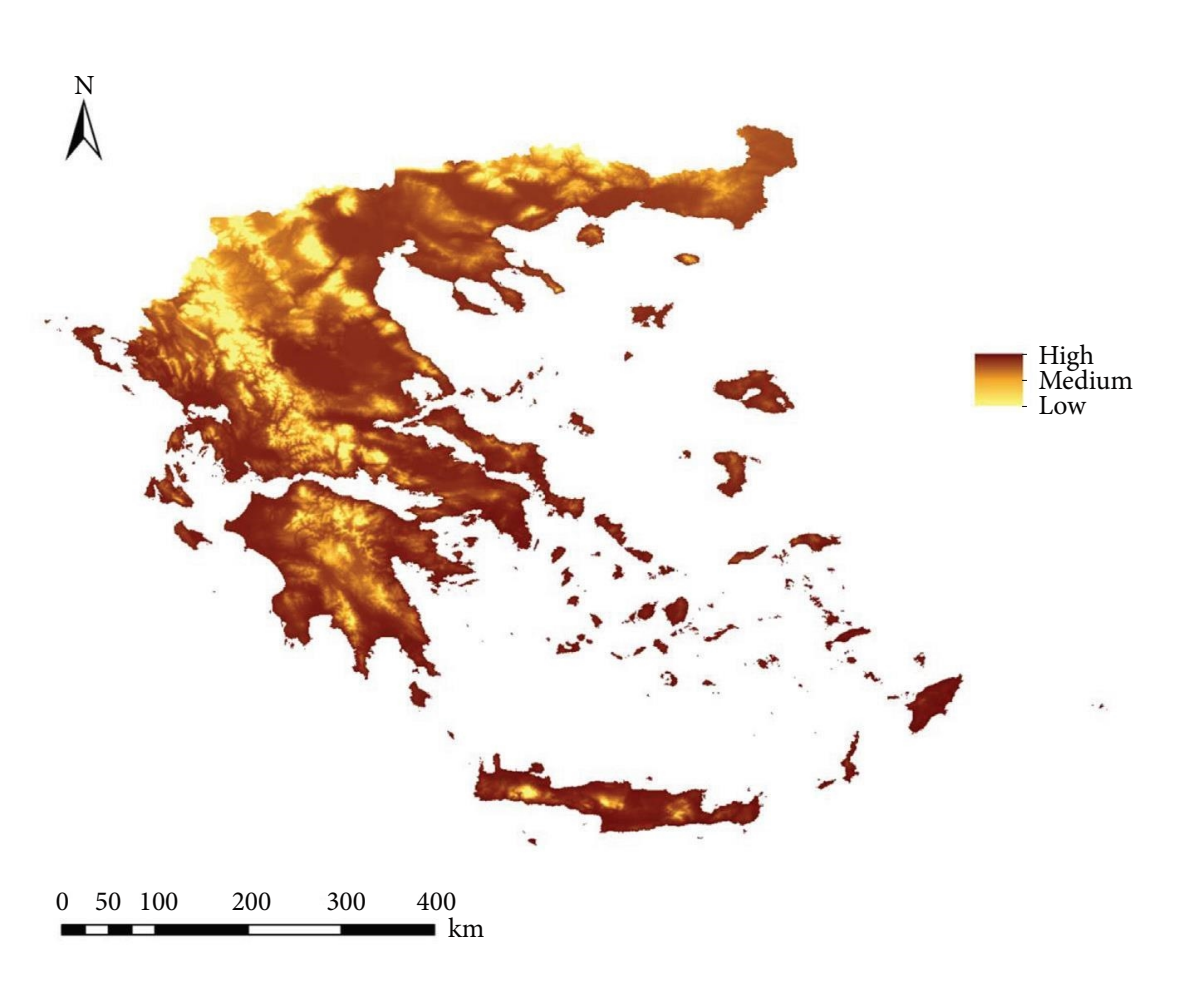
Prediction of *Phlebotomus perfiliewi* and *Ph. tobbi* infection rate in Greece.

Figure 4 represents the map of the *L. infantum* infection rate in both vectors in Greece. Areas with higher infection rates correspond to low-altitude territories and coastal zones, such as Macedonia, Thessaly, Attica, the coasts of the Peloponnese, and islands in both the Aegean and Adriatic Seas.

### 3.3. Risk Maps and Validation

**Figure 5:**
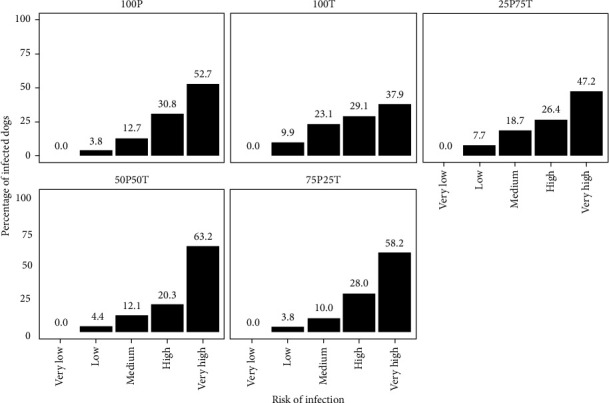
Regression plot for the validation of the ecological niche model between the risk of infection and the percentage of geolocated infected dogs reported by Symeonidou et al. [6].

**Figure 6:**
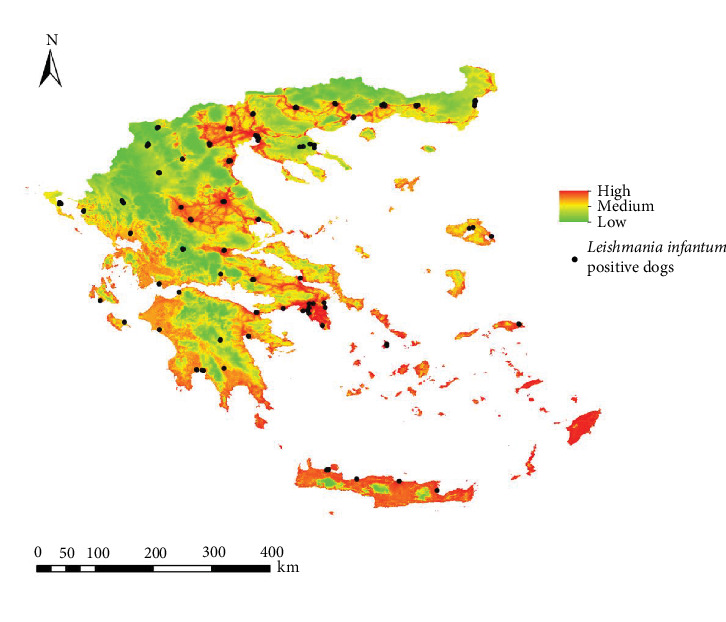
Potential transmission risk map of *Leishmania infantum* in Greece (50P-50T) resulting from the validation with the best histogram, with infected dogs superimposed over the maps.

After generating fitness models for both vectors with different contributions (0-100, 25-75, 50-50, 75-25, 100-0), weighted by infection rate, we obtained five risk maps of potential *L. infantum* infection in Greece. The histogram for the risk map with the 50P-50T contribution (50% *Ph. perfiliewi* − 50% *Ph. tobbi*) (Figure 5) best estimated the percentage of geolocated infected dogs in very high risk areas (63.2%) (Figure 6).

**Figure 7:**
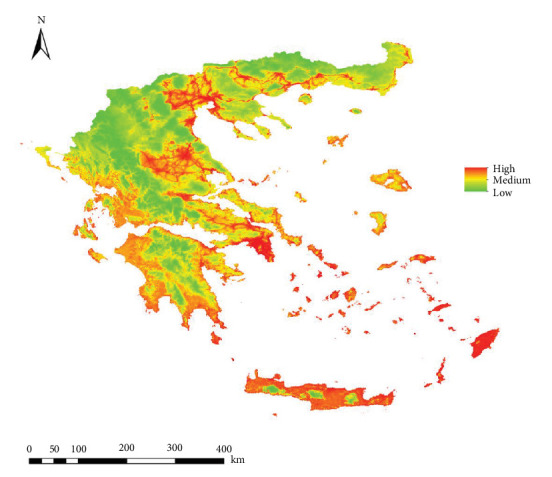
Potential transmission risk map of *Leishmania infantum* in Greece (50P-50T) resulting from the validation with the best histogram.

The chosen risk map (50P-50T) presents a higher risk of infection in urban, coastal, and low-altitude territories. Central Macedonia, Thessaly, Attica, and islands such as the Cyclades, Dodecanese and Crete, as well as some urban areas of Thrace close to the coast, present a high risk of infection. The coasts of both Epirus and Peloponnese have a medium risk, and in the cooler, higher altitude inland areas of the continent, the risk is low (Figure 7).

### 3.4. Future Projections of Transmission Risk

**Figure 8:**
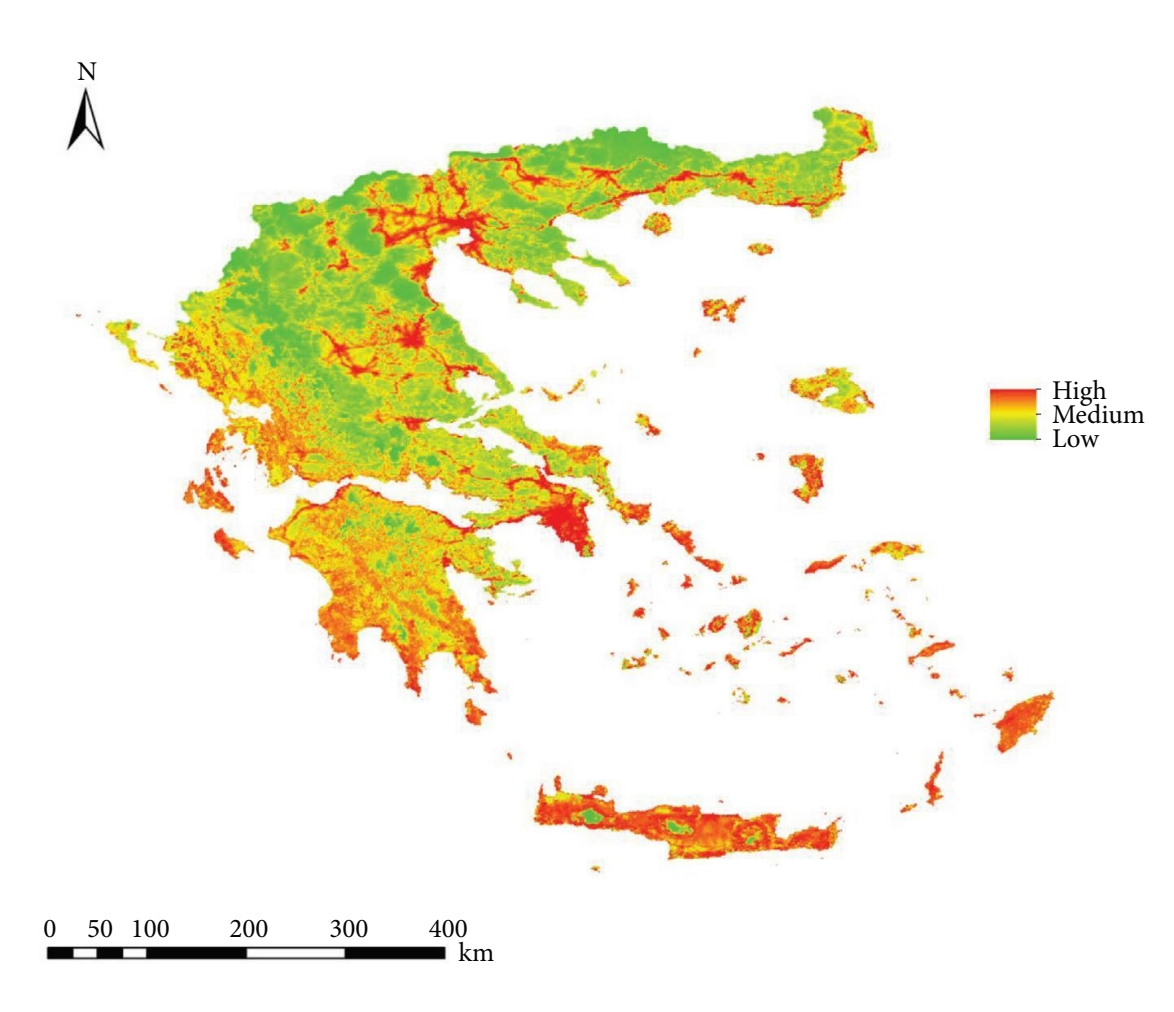
Projections of the transmission risk of *Leishmania infantum* for 2080 in Greece under the climate change scenario RCP 8.5.

**Figure 9:**
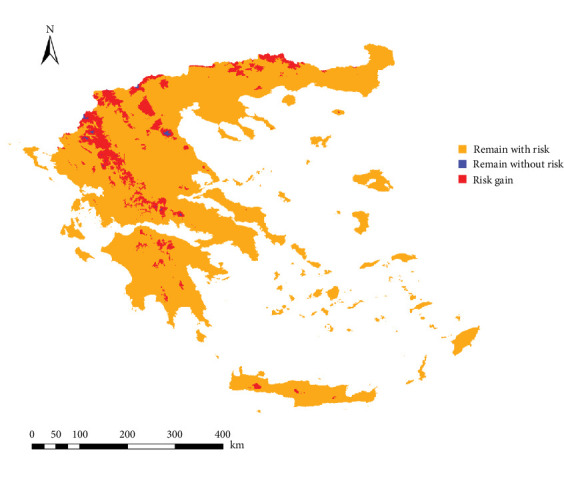
Range change analysis showing areas of gain, loss, and those that remain as in the present of the infection risk of *Leishmania infantum* infection in Greece.

The projected model for 2080 on *L. infantum* transmission risk showed an increase in infection risk in Epirus, the Peloponnese Peninsula (mainly in its interior regions), and the Aegean islands. Additionally, the risk increase extends to higher-altitude areas (Figure 8). In the range change analysis (Figure 9), most of the infection risk gain (marked in red) occurs in the western territories of the country and towards higher altitudes, increasing by 9.6% compared to the current risk. In the rest of the territories, the risk remains unchanged (marked in blue and orange).

## 4. Discussion

The objective of this study is to predict, for the first time, the potential risk of *L. infantum* infection in Greece by modeling the ecological niche of two disease vectors (*Ph. perfiliewi* and *Ph. tobbi*) and calculating the parasite infection rate in sandflies. The selection and validation method allowed us to choose the best model from a set of candidates with different contributions from both sandfly species, selecting the one that most accurately reflected reality.

Prior to this study, the ENM methodology had already been applied to determine the distribution of leishmaniasis in both Europe and other regions. Most of these studies used only presence data of infected hosts or vectors [14–23]. In Greece, there are only two local-level studies: one used only GIS tools, while the other performed an ENM using individual records of infected hosts (dogs and humans) [24, 25].

Our risk map combines the ecological niche of *Ph. perfiliewi* and *Ph. tobbi* (including widespread occurrences until present), the most widely distributed species in Greece, and the calculation of the *L. infantum* infection rate in the vector, thereby achieving a more realistic model of disease risk. The fact that Rioux et al. [4] estimate the infection rate of *Leishmania infantum* in *Phlebotomus ariasi* in France should not have a big influence on the rate suffered by other closely related species in Mediterranean countries, where the disease is widespread, as it has already been applied [23]. This weighting strategy, as seen in previous studies, improves the predictive power of the resulting model [23, 30].

The variables that contributed the most to explaining the potential distribution of sandflies were Human Footprint (built environment, population density, electric power infrastructure, farmland, grazing land, roads, railways, and waterways) and BIO4 (temperature seasonality) for *Ph. perfiliewi*, and BIO1 (annual mean temperature) and Human Footprint for *Ph. tobbi*. Regarding Human Footprint, areas with high human presence constitute an ideal habitat for the maintenance of sandfly populations [45]. Locations such as parks and agricultural lands provide a large number of *L. infantum* reservoirs, including rats, rabbits, and cats, which facilitate the establishment of the canine leishmaniasis biological cycle and can occasionally trigger human leishmaniasis outbreaks [45–51].

On the other hand, temperature-related variables (BIO4 for *Ph. perfiliewi* and BIO1 for *Ph. tobbi*) have a positive effect on the ecology and biology of sandflies, being essential for egg production rates, juvenile development, number of generations per year, activity period, and adult survival [52, 53]. Another important variable, though with less influence in our models, is BIO12 (precipitation seasonality). This factor affects habitats identified as suitable for hares and other parasite reservoirs (natural grasslands, rainfed agricultural lands, and areas with natural vegetation), which exhibit moderate to high annual precipitation [54].

In this study, we weighted the models to obtain different combinations and determined that, among all, the 50P-50T combination best reflects the potential infection risk. The validation performed with 175 georeference *L. infantum*-positive widespread dogs allowed to test the risk model with a moderate number of animals tested. Increasing the sample resolution in future might help to verify all potential high-risk areas. The high-risk areas predicted in this model coincide with zones of high human pressure and temperature, where dogs and humans live in close proximity, potentially increasing the risk of infection for susceptible individuals [6]. Conversely, mountainous areas at higher altitudes and with lower temperatures, which reduce both vector habitat suitability and infection rates, show risk values close to zero.

Regarding the 2080 projection, an increase in infection risk (by 9.6%) is observed in higher-altitude areas, potentially driven by global warming. However, no decrease in risk is noted in any part of the country, as it remains stable in areas where no increase occurs. This study predicts the displacement of canine leishmaniasis towards higher altitudes and its colonization of areas where it was previously absent [12, 22, 29, 55, 56].

## 5. Conclusion

We have been able to map the risk of *L. infantum* infection in dogs and potentially in humans by weighing two potential vectors and calculating the infection rate under both current and future conditions. Future epidemiological studies focusing on smaller regions (where presence points are typically collected from urban and rural areas) and incorporating both presence and absence data could enable the development of more precise risk models than those proposed here [57]. At the same time, to validate these models, it is important to use additional infected organisms and continue incorporating the ecological niches of all species involved in parasite transmission. In doing so, increasingly realistic models can be developed, providing valuable tools for veterinary and public health professionals in disease prevention and control.

## Figures and Tables

**Table 1:** Percent contribution of the variables selected in the ecological niche model for *Phlebotomus perfiliewi* and *Ph. tobbi*.

Variable	Percent contribution *Phlebotomus perfiliewi*	Percent contribution *Phlebotomus tobbi*
BIO_1_	5.59	33.17
BIO_2_	9.36	21.1
BIO_4_	11.51	6.27
BIO_12_	7.57	8.92
Human footprint	55.65	23.12
Herbaceus	3.87	3.6
Shrubs	6.45	3.82

## Data Availability

The data that support the findings of this study are available from the corresponding author upon reasonable request.
